# Impact of COVID-19 on Pregnancy

**DOI:** 10.7150/ijms.49923

**Published:** 2021-01-01

**Authors:** Chiu-Lin Wang, Yi-Yin liu, Chin-Hu Wu, Chun-Yu Wang, Chun-Hung Wang, Cheng-Yu Long

**Affiliations:** 1 Department of Obstetrics and Gynecology, Kaohsiung Municipal Siaogang Hospital, Kaohsiung Medical University, Kaohsiung, Taiwan; 2 Department of Obstetrics and Gynecology, Kaohsiung Medical University Hospital, Kaohsiung, Medical University, Kaohsiung, Taiwan; 3 Graduate Institute of Medicine, College of Medicine, Kaohsiung Medical University, Kaohsiung, Taiwan. urolong@yahoo.com.tw; 4 Advanced mechanical engineering with management MSc, School of Engineering, University of Leicester, United Kingdom; 5 Department of Nursing, Fooyin University, Ta-Liao District, Kaohsiung, Taiwan

**Keywords:** Coronavirus disease 2019 (COVID-19), Severe acute respiratory syndrome coronavirus 2 (SARS-CoV-2), Wuhan pneumonia, Pregnancy

## Abstract

Coronavirus disease 2019 (COVID-19) is caused by severe acute respiratory syndrome coronavirus 2 (SARS-CoV-2) and is an emerging disease. There has been a rapid increase in cases and deaths since it was identified in Wuhan, China, in early December 2019, with over 4,000,000 cases of COVID-19 including at least 250,000 deaths worldwide as of May 2020. However, limited data about the clinical characteristics of pregnant women with COVID-19 have been reported. Given the maternal physiologic and immune function changes during pregnancy, pregnant women may be at a higher risk of being infected with SARS-CoV-2 and developing more complicated clinical events. Information on severe acute respiratory syndrome (SARS) and Middle East respiratory syndrome (MERS) may provide insights into the effects of COVID-19's during pregnancy. Even though SARS and MERS have been associated with miscarriage, intrauterine death, fetal growth restriction and high case fatality rates, the clinical course of COVID-19 pneumonia in pregnant women has been reported to be similar to that in non-pregnant women. In addition, pregnant women do not appear to be at a higher risk of catching COVID-19 or suffering from more severe disease than other adults of similar age.

Moreover, there is currently no evidence that the virus can be transmitted to the fetus during pregnancy or during childbirth. Babies and young children are also known to only experience mild forms of COVID-19. The aims of this systematic review were to summarize the possible symptoms, treatments, and pregnancy outcomes of women infected with COVID-19 during pregnancy.

## Introduction

In December 2019, cases of a novel coronavirus-associated pneumonia were first reported in Wuhan City, China 1. It has since been prevalent in China and in other countries all over the world. This new coronavirus disease was termed Coronavirus Disease 19 (COVID-19) by the World Health Organization (WHO) on February 11, 2020, and it was declared a pandemic on March 11, 2020. The International committee on Taxonomy of Viruses renamed the virus from 2019 novel coronavirus (2019-nCoV) to severe acute respiratory syndrome coronavirus 2 (SARS-CoV-2).

Coronaviruses are enveloped, non-segmented, single-stranded ribonucleic acid (RNA) viruses causing diseases from common colds to severe fetal diseases. The two best known fetal viruses are SARS-CoV, which causes severe acute respiratory syndrome (SARS), and MERS-CoV, which causes Middle East respiratory syndrome (MERS). The genome of SARS-CoV-2 shares about 80% and 50% similarity with SARS-CoV and MERS-CoV, respectively 2.

Pregnant women may be susceptible to developing more severe symptoms after infection with respiratory viruses, due to physiological changes of the immune and cardiopulmonary systems during pregnancy 3. Both SARS-CoV and MERS-CoV have been associated with higher case fatality rates and more severe complications during pregnancy 4,5. However, there are limited reports on the impact of COVID-19 during pregnancy. As a result, the potential effects on fetal and neonatal outcomes are unclear, and studies are urgently needed with regards to the management of pregnant women with COVID-19. The objective of this study was to review the clinical manifestations, maternal and perinatal outcomes of COVID-19 during pregnancy.

### Clinical manifestations

The most common clinical symptoms of COVID-19 in the general population are fever (91%), cough (67%), fatigue (51%), and dyspnea (30%) 6. Fever (68%) and cough (34%) are also the most common symptoms in pregnant women with COVID-19, with other symptoms including dyspnea (12%), diarrhea (6%), and malaise (12%) 7. These clinical manifestations are similar to those in nonpregnant women 8,9.

According to the severity of disease, COVID-19 is classified as being mild (symptomatic or mild pneumonia), severe (tachypnea≧30 breaths/min, or oxygen saturation ≤93% at rest, or PaO2/FiO2 <300 mmHg), and critical (respiratory failure requiring endotracheal intubation, shock, or other organ failure that requires intensive care), accounting for 81%, 14% and 5% of cases in the general population, respectively 10. World Health Organization reported a large cohort study of 147 pregnant women with COVID-19, only 8% and 1% were severely and critically ill, respectively 11. This suggests that most pregnant women with COVID-19 have milder symptoms compared with the general population. Another study also reported that pregnant women with COVID-19 pneumonia had milder disease and good recovery 12.

Severe COVID-19 has been widely reported in older adults (>60 years), the immunosuppressed and those with comorbidities such as diabetes, hypertension and chronic lung disease^13^,^14^. Most pregnant women are younger than middle age; however, it is important to consider the potential impact of pre-existing hyperglycemia and hypertension on the outcomes of COVID-19 in pregnant women.

In pregnant women who develop COVID-19 pneumonia, early data have shown a similar rate of intensive care unit (ICU) admission to the nonpregnant women, but higher rates of preterm and cesarean delivery. The case fatality rates of SARS and MERS were 25% and 27%, respectively, compared to only 1% for COVID-19.

However, thus far, COVID-19 appears to result in less severe maternal pregnancy outcomes than SARS or MERS 14.

Despite the limited data about COVID-19 during pregnancy, information on illnesses associated with other highly pathogenic coronaviruses SARS and MERS provide insights into the effect of COVID-19 during pregnancy.

### Diagnosis

A real-time reverse transcriptase polymerase chain reaction (RT-PCR) assay is the gold standard for diagnosis. Chest X-ray (CXR) and chest computed tomography (CT) may aid in the diagnosis, and can be used to assess the extent and follow-up of COVID-19. CXR can be rapidly and easily performed at the bedside, whereas chest CT is more sensitive in the early stage of infection. However, concerns regarding the potential teratogenic effects to the fetus from radiation exposure are unavoidable. The accepted cumulative dose of ionizing radiation during pregnancy is 5 rad, and no single diagnostic study exceeds this dose. The amount of exposure to the fetus from a two-view CXR of the mother is only 0.00007 rad, and 10 chest CT slices result in an exposure of <0.1 rad 15. Therefore, in pregnant women with suspected COVID-19, CXR and CT if indicated can be considered and performed safely. An abdominal irradiation shield over the gravid uterus can also be used for further fetal protection. Pulmonary ultrasound has also been suggested for a quick diagnosis of pneumonia in pregnant women 16.

### Fever

Fever is the most common manifestation in patients with COVID-19, and it may be associated with an increased risk of congenital anomalies, including neural tube defects and miscarriage during organogenesis in the first trimester. If prescribing antipyretic agents, non-steroidal inflammatory drugs (NSAIDs) must be avoided, since they have been associated with a higher risk of miscarriage in early pregnancy and fetal pulmonary hypertension after 30 weeks' gestation. Acetaminophen can be prescribed safely and may lower the risks during pregnancy associated with fever exposure.

### Maternal outcomes

#### First trimester

Li et al. reported that four of seven pregnant women with SARS presenting in the first trimester had spontaneous miscarriages, and two had elective terminations. The only newborn survivor was delivered at term, and no anomalies were reported 17.

There is only one report of a pregnant woman with MERS, who presented at 6 weeks' gestation. She was asymptomatic and subsequently delivered a healthy infant at term 18. There are currently no data on first-trimester COVID-19 infections hence the effect of COVID-19 on the fetus in the first trimester is unknown.

#### Second/third-trimester

Lam et al. suggested that pregnant women with SARS had higher rate of maternal mortality, intubation and ICU admission than nonpregnant women with SARS, but that transmission of the virus to the infant did not occur 19. More complications such as miscarriage, preterm delivery, and small for gestational age neonates, have also been reported. However, one study reported no increases in the risks of spontaneous abortion and spontaneous preterm birth in pregnant COVID-19 women 20.

Many systemic infections and inflammatory states are associated with preterm birth, and preterm births in women infected with COVID-19 have been reported. However, most of them were reported in China which has a different medical system, and it is not yet clear whether SARS-CoV-2 caused these premature births or whether it was iatrogenic due to maternal or fetal distress or other indications.

### Medical treatments

Antiviral agents - There are currently no approved treatments for COVID-19, although several agents are being evaluated, including chloroquine and remdesivir. Hydroxychloroquine can cross the placenta and it is secreted in milk, however its concentration is low so that no fetal ocular toxicity has been reported. It is therefore used to treat systemic lupus erythematous in pregnant women. Other antiviral agents are not recommended in pregnancy unless they are part of a clinical trial.

Antenatal corticosteroids -Corticosteroids may delay virus clearance from the body, and they are associated with increased risks of morbidity and mortality in patients with COVID-19 21. However, antenatal corticosteroids between 24+0 and 33+6 weeks of gestation to promote fetal lung maturation is recommended by American College of Obstetricians and Gynecologists (ACOG), in order to reduce neonatal morbidity and mortality from preterm birth in pregnant women with COVID-19. These recommendations must be tailored to specific clinical circumstances, weighing the individualized risks and benefits to both neonates and mothers.

Magnesium sulphate -Magnesium sulphate is used for neonatal neuroprotection and eclampsia prophylaxis. The benefits of therapy should be weighed against potential risks of maternal respiratory depression.

### Delivery

Several studies have reported no evidence of SARS-CoV-2 in umbilical cord blood, placenta, and/or amniotic fluid 22,23. As there has been no evidence of vertical transmission of COVID-19 24,25, COVID-19 is not an indication for cesarean section. The mode and timing of delivery should be individualized based on the severity of disease, existing comorbidities and obstetric indications. Nonetheless, the babies of most pregnant women with COVID-19 have been reported to be delivered by cesarean section. However, most of these studies were reported in China, where public health is very different, and the reasons for fetal distress were not clearly discussed. Therefore, it is unclear whether the indications for cesarean section were due to poor maternal conditions and fetal hypoxia caused by maternal COVID-19 or other causes. Further research is needed to assess the risk and to produce guidelines for delivery times and methods in pregnant COVID-19 women.

There is no evidence regarding whether delayed cord clamping increases the risk of transmitting pathogens from mother to newborn even in cases of maternal infection via direct contact 26. However, early cord clamping may minimize the risk of viral transmission by avoiding longer, close contact with the infected mother.

Both regional anesthesia and general anesthesia can be considered, depending on the clinical condition of the patient 27. However, regional anesthesia is preferable in order to lower the risk to the staff. Since the vernix caseosa contains antimicrobial peptides, we recommend leaving it in place until 24 h after birth 28.

### Newborns

In a retrospective cross-sectional study, 45 newborns were born to mothers with SARS-CoV-2, only 3 (6.6%) were tested positive by throat swab. However, they were all asymptomatic and the test became negative later, which was suggesting transient colonization 29. In a review of 836 newborns of COVID-19 mothers, 35 newborns (4.2%) tested positive via polymerase chain reaction (PCR) and most of them were reported no respiratory or other illness. About 22% of newborns were born prematurely, only on severely and critically ill mothers 30. However, whether they were iatrogenic caused, or as a result of acute respiratory distress and other severe complications in infected women is not yet clear. Walker et al review 666 newborns from 655 infected mothers, 8/292 (2.7%) born vaginally were tested positive compared with 20/374 (5.3%) born by Caesarean section 31.

### Breast feeding

A previous study reported that all samples of breast milk from 26 infected women tested negative for SARS-CoV-2 32. However, droplet transmission can occur through close contact during breastfeeding. Mothers are encouraged to practice excellent hand hygiene and pump their breasts following birth to initiate lactogenesis. Infants should be fed pumped milk by another healthy caregiver until the mother has recovered. Mothers who wish to breastfeed directly are encouraged to practice excellent hand hygiene and wear a surgical mask during breastfeeding, since an infected mother can transmit the virus through respiratory droplets during breastfeeding.

## Discussion

There are limited data on the impact of COVID-19 on pregnant women and their babies. All studies included in this review were case reports or series of low quality. Compared with SARS and MERS, COVID-19 appears to be less lethal. Most of the pregnant women with COVID-19 in this review were asymptomatic or only had mild symptoms, however particular attention should be given to those with underlying diseases, since they are at a higher risk of developing severe disease as with the general population.

Pregnant women with SARS have been reported to have a high miscarriage rate. Hence, an increased risk of miscarriage in women with COVID-19 cannot be ruled out at this stage, due to the lack of data on first-trimester COVID-19 infection. In women with COVID-19 and ongoing pregnancy, surveillance for fetal growth restriction is reasonable, given that fetal growth restriction was observed in most ongoing pregnancies with SARS.

In women with SARS and MERS, cesarean section was most commonly indicated due to maternal hypoxemia. As COVID-19 maternal illness does not appear to be as severe as SARS and MERS, the high rate of cesarean section (almost all) is unreasonable, and further analysis is needed.

There is currently no evidence of vertical transmission for any other coronavirus. In addition, SARS-CoV-2 has not been found in the amniotic fluid, umbilical cord blood, neonatal throat swabs or breast milk. The mode of delivery should therefore depend on obstetric indications and not on COVID-19.

The current knowledge about COVID-19 is limited, and it shares both similarities and differences with SARS and MERS. Careful monitoring of both mother and fetus, and measures to prevent neonatal infection are warranted during the COVID-19 pandemic.
